# Supplementary material for: Detecting genuine multipartite entanglement in multi-qubit devices with restricted measurements
Source: Nat Commun. 2026 Feb 17;17:1707. doi: 10.1038/s41467-026-69320-4 (PMC12914044; doi:10.1038/s41467-026-69320-4)
Supplement: Supplementary file 1 — Supplementary Information [file 41467_2026_69320_MOESM1_ESM.pdf]

Supplementary Information for "Detecting genuine multipartite entanglement in multi-qubit devices with restricted measurements"

Nicky Kai Hong Li [1,2,3,*] Xi Dai [4,5,†] Manuel H. Muñoz-Arias [6]
Kevin Reuer [4,5] Marcus Huber [1,2,3] and Nicolai Friis [1,2,‡]

[1] *Technische Universität Wien, Atominstitut, Stadionallee 2, 1020 Vienna, Austria*
[2] *Vienna Center for Quantum Science and Technology, TU Wien, 1020 Vienna, Austria*
[3] *Institute for Quantum Optics and Quantum Information (IQOQI), Austrian Academy of Sciences, Boltzmanngasse 3, 1090 Vienna, Austria*
[4] *Department of Physics, ETH Zurich, CH-8093 Zurich, Switzerland*
[5] *Quantum Center, ETH Zurich, CH-8093 Zurich, Switzerland*
[6] *Institut Quantique and Département de Physique, Université de Sherbrooke, Sherbrooke J1K 2R1 QC, Canada*

(Dated: January 27, 2026)

CONTENTS

Supplementary Note 1. Review of previous methods 1
Supplementary Note 2. Proof of Propositions 1 and 2 2
Supplementary Note 3. Proof of Observation 1 3
Supplementary Note 4. Algorithm for calculating the criterion upper bound 3
Supplementary Note 5. Reducing the number or weight of required stabilizers with local Clifford transformations 4
Supplementary Note 6. A brief review of SDP 5
Supplementary Note 7. Theoretical examples 6
a. Graphs requiring $\leqslant$4-body correlators in $\mathcal{W}_G^\gamma$ 6
b. Graphs requiring $4 < m \leqslant O(1)$-body correlators in $\mathcal{W}_G^\gamma$ 7
c. Star graphs and complete graphs 8
d. Noisy Dicke states 8
Supplementary Note 8. Measuring Pauli observables of microwave photons 8
Supplementary Note 9. Simulating graph-state generation 9
Supplementary Note 10. Error analysis of certifying GME/k-inseparability 10
Supplementary Note 11. More simulation results 11
References 12

Supplementary Note 1. Review of previous methods

To provide some context for the methods we derive here, let us briefly review some of the methods that have previously been used to detect multipartite entanglement, particularly for states close to graph states. The main idea behind most of these witnesses/criteria is to construct a suitable linear witness, that is, a Hermitian operator W such that $\mathrm{Tr}(W\rho) \geqslant 0$ for all k-separable ρ and $\mathrm{Tr}(W\sigma) < 0$ for at least one k-inseparable state σ. For example, $W = \frac{1}{2}\mathbb{1} - |\mathrm{GHZ}_n\rangle\langle\mathrm{GHZ}_n|$, where $|\mathrm{GHZ}_n\rangle = \frac{1}{\sqrt{2}}(|0\rangle^{\otimes n} + |1\rangle^{\otimes n})$, is a GME witness for all n-qubit states since $\langle\mathrm{GHZ}_n|\rho|\mathrm{GHZ}_n\rangle \leqslant \frac{1}{2}$ for any 2-separable state ρ [1]. By the supporting-hyperplane theorem (or the more general Hahn-Banach theorem), there exists a hyperplane (with a corresponding witness operator W) that separates any k-inseparable state from the set of k-separable states. For witnesses constructed using the stabilizer formalism, the witness operator W is typically chosen to be some linear combination of a subset of a graph state's stabilizers. To achieve optimal performance with this type of witnesses, the underlying graph state should be the one that is closest to the quantum state for which one wants to certify entanglement.

As we will see in this section, most witnesses in the literature require the measurement of up to $O(n)$-body observables, which makes them infeasible to implement in experiments that can only measure at most $O(1)$-body observables at a time (see, e.g., Ref. [2] and Sec. II D). We also emphasize that previous studies we are aware of primarily focus on minimizing the number of local measurement settings [3], whereas our focus in this paper is on limiting the number of particles involved in each measured observable. Furthermore, many existing witnesses cannot distinguish different levels of k-inseparability for $2 \leqslant k \leqslant n$ (i.e., they can only tell if the state is GME or not). For moderately noisy states, such witnesses may discard useful information about the entanglement structure if they cannot tell apart, for instance, fully separable and tri-separable states, even though the latter may still possess significant entanglement.

We begin by reviewing several well-known witnesses based on the stabilizer formalism. In Refs. [1, 4], a family of witnesses was proposed to detect GME or to rule out full separability. In general, these witnesses require measuring $O(n)$-body stabilizers, with the exception of one particular type of GME witness, see [4, Eq. (45)], which does not detect k-inseparability that is non-GME. In Sec. II B, we show that this specific type of GME witness is a special case (and also corresponds to the worst-case bound) of a broader family of GME and k-inseparability criteria proposed in this work. Some of the witnesses in Refs. [1, 4] have since been used to cer-

* kai.li@tuwien.ac.at
† xi.dai@phys.ethz.ch
‡ nicolai.friis@tuwien.ac.at

tify GME in experimentally generated GHZ and cluster states of up to 14 photons/qubits [5], as well as in ring-graph and certain tree-graph states of up to 8 qubits [6]. Subsequently, Ref. [7] introduced a family of general partition-inseparability (including GME and k-inseparability) witnesses that depend on the chromatic number of the underlying graph corresponding to the graph state. For cluster states, which correspond to 2-colorable graphs, the required measurements involve up to $\lceil \frac{n}{2} \rceil$-body stabilizers. For general graph states, the witnesses also need measurements of up to $O(n)$-body stabilizers.

Another class of witnesses for multipartite entanglement is based on the positive partial-transpose (PPT) criterion [8, 9]. For example, Refs. [10, 11] proposed a variety of fully decomposable and fully PPT witnesses that detect GME (but not general k-inseparability), with the optimal ones obtained via SDP. Interestingly, the optimal fully decomposable witnesses for graph states can be constructed solely from their stabilizers. The same references also provide stabilizer-based witnesses that do not require optimization. In general, these witnesses require measuring up to n-body observables. Building on this idea, Ref. [12] demonstrated that fully decomposable GME witnesses can be found using SDP optimization with a large set of 2-body observables. In principle, this method can be extended to include higher-body observables. However, such SDP optimizations become computationally infeasible (in both memory and runtime) for large particle numbers n, due to the exponential growth of the Hilbert-space dimension with n.

An alternative approach to witnessing multipartite entanglement involves measuring an observable whose expectation value provides a lower bound on the fidelity with respect to a target GME graph state. If this fidelity lower bound exceeds a certain threshold (e.g., $F(\rho_{\text{GME}}, |\text{GHZ}_n\rangle) > 0.5$, as discussed before), the state is certified to be GME/k-inseparable. Reference [13] shows how to construct such lower bounds using carefully chosen local measurements and establishes their connection to GME witnesses. This technique improves the noise tolerance of certain witnesses from Refs. [1, 4] by introducing different positive-operator relaxations and local filters. These methods have been applied in several experiments to lower bound state fidelities and certify GME in various graph states [5, 14]. Although the fidelity bounds and associated GME witnesses introduced in this work do not require many local measurement settings, they can still involve observables that act on all qubits.

From the perspective of error mitigation, Ref. [15] improved upon the methods of Refs. [1, 4] for lower bounding the fidelities of cluster states by incorporating specific additional stabilizer terms into the fidelity-estimation observable. These extra terms are designed to address many of the experimentally relevant Pauli errors, from low- to high-order, thereby tightening the estimated fidelity lower bound for cluster states while keeping the number of local measurement settings linear in the number of qubits. This method also requires measuring observables that act on up to n qubits and is tailored specifically for cluster states (and potentially other 2-colorable graph states).

Although not originally developed for entanglement certification, Ref. [16] introduced an efficient method for estimating the fidelity between a pure state $|\psi\rangle$ and an arbitrary state ρ by measuring subsets of Pauli operators sampled according to the Pauli distribution of $|\psi\rangle$, i.e., $p(k) = \frac{1}{d}|\langle\psi|P_k|\psi\rangle|^2$ where the P_k for $k \in \{0, 1, \ldots, d^2-1\}$ are the (tensor products of) Pauli operators (including the identity $P_0 = \mathbb{1}$). For graph/stabilizer states $|\psi\rangle$, only 2^n stabilizer terms need to be sampled, as all other Pauli terms have zero coefficients. However, with this approach, one cannot restrict to measuring only up to $O(1)$-body Paulis.

There also exist GME and k-inseparability witnesses that are independent of the stabilizer formalism. These are based on inequalities that depend on specific diagonal and off-diagonal elements of the density matrix, where these elements are related by partition-specific permutations [17–19]. Accessing these off-diagonal elements typically requires measuring n-body observables (see, e.g., Refs. [13, 19]), rendering such witnesses impractical in the experimental scenarios considered in this work.

Furthermore, there exist GME and k-inseparability criteria based on the Ky Fan norms of different index-permuted density matrices (Theorem 3 of Ref. [20]), which require full state tomography to evaluate exactly. In the same work, they also propose methods to lower bound these norms via convex optimizations constrained by various permutation moments, which can be estimated using shadow tomography [21, 22], randomized measurements [23–25], or a hybrid protocol—each using only one fresh copy of the state per measurement round. However, such lower bounds are generally loose, and applying these methods requires measuring at least $2^n - 2$ permutation moments (each requiring $O(2^{1.187n})$ measurements) and solving $2^{n-1}-1$ optimization problems, resulting in substantial measurement and computational overhead. In contrast, our method requires measuring at most $O(n^2)$ of the graph-state stabilizers and, when using the looser bound, only a single optimization over the parameter γ.

Finally, we highlight several witnesses that are state-independent and require only simple measurement observables and settings. Reference [26] proposed an optimizable, single-parameter family of k-inseparability witnesses, which were used to study the entanglement structure of various 8-photon states in their experiment. These witnesses rely on measurements of full n-body observables $X^{\otimes n}$ and $Z^{\otimes n}$. In contrast, the entanglement witnesses from Ref. [27] involve only 2-body observables—$X^{\otimes 2}$, $Y^{\otimes 2}$, and $Z^{\otimes 2}$—acting on different pairs of qubits, and were successfully used to detect GME in a system of 20 trapped-ion qubits. However, these witnesses are limited in that they do not detect non-GME k-inseparability and are ineffective for high-fidelity graph states.

Supplementary Note 2. Proof of Propositions 1 and 2

In this appendix, we will prove Propositions 1 and 2 which are used in the proof of Theorem 1.

Proposition 1. *Let $\{A_i\}_{i=1}^m$ and $\{B_i\}_{i=1}^m$ be subsets of orthonormal self-adjoint bases of $d_1 \times d_1$ and $d_2 \times d_2$ complex matrices, respectively, such that $\{A_i, A_j\} = 2\delta_{ij}\mathbb{1}_{d_1}$ and*

$\{B_i, B_j\} = 2\delta_{ij}\mathbb{1}_{d_2}$ *for all* i, j. *Then, the expectation values of* A_i *and* B_i *with respect to any quantum states* $\rho \in \mathcal{D}(\mathbb{C}^{d_1})$ *and* $\sigma \in \mathcal{D}(\mathbb{C}^{d_2})$ *must satisfy* $\sum_{i=1}^m |\langle A_i\rangle_\rho \langle B_i\rangle_\sigma| \leqslant 1$.

Proof. By the Cauchy-Schwarz inequality and Lemma 2,

$$\sum_{i=1}^m |\langle A_i\rangle\langle B_i\rangle| \leqslant \sqrt{\sum_{i=1}^m \langle A_i\rangle^2} \cdot \sqrt{\sum_{i=1}^m \langle B_i\rangle^2} \leqslant \sqrt{\max_i\{\langle A_i^2\rangle\}} \cdot \sqrt{\max_i\{\langle B_i^2\rangle\}} \leqslant 1, \tag{S1}$$

where we omit the subscripts ρ and σ. The last inequality follows from the fact that $A_i^2 = \mathbb{1}_{d_1}$, $B_i^2 = \mathbb{1}_{d_2}$ $\forall$ i, and $\{A_i, A_j\} = \mathbf{0}_{d_1}$, $\{B_i, B_j\} = \mathbf{0}_{d_2}$ $\forall$ $i \neq j \Rightarrow \mathcal{K} = 0$. □

Proposition 2. *Any* k*-cut of a connected graph must remove at least* $k-1$ *edges that are shared among at least* k *vertices.*

Proof. Any k-partition of a graph must have at least one vertex in each partition. Consider a representative graph which replaces all vertices of the full graph in each partition with one vertex and all edges that connect two different partitions with an edge between the two corresponding representative vertices. For the full graph to be connected, the k vertices in the representative graph must be connected as well and the minimal number of edges in any connected k-vertex graph is $k-1$. □

Supplementary Note 3. Proof of Observation 1

In this appendix, we provide the full details for the examples where the optimal criterion for r-inseparability in Eq. (6) is achieved with $\gamma \in (0,1)$. First, we observe that all Cthulhu graphs parametrized by the integer $r \geqslant 3$ (see Fig. 2) has two different optimal r-cuts associated to $\gamma \leqslant \big(\lfloor\frac{r}{2}\rfloor - 1\big)/\lfloor\frac{r}{2}\rfloor =: \gamma_r^*$ and $\gamma \geqslant \gamma_r^*$. As illustrated in Fig. S1, the two optimal r-cuts correspond to r-partition subgraphs $\overline{G}_-^{(r)}$ and $\overline{G}_+^{(r)}$, which have different number of vertices $|\overline{V}_\pm^{(r)}|$ and edges in the maximum-cardinality matching $|\overline{E}_{\pm,\mathrm{mcm}}^{(r)}|$. Hence, the reduction term $R_r^\gamma := \min_{\text{all } r\text{-cuts}}\big(\gamma|\overline{V}^{(r)}| + (1-\gamma)|\overline{E}_{\mathrm{mcm}}^{(r)}|\big)$ for the full range of $\gamma \in [0,1]$ takes the following form

$$R_r^\gamma = \begin{cases} r\gamma + 1 & \text{for } \gamma \leqslant \gamma_r^*, \\ \lceil\frac{r}{2}\rceil\gamma + \lfloor\frac{r}{2}\rfloor & \text{for } \gamma \geqslant \gamma_r^*. \end{cases} \tag{S2}$$

Now, suppose that $\rho = \frac{p}{2^n}\mathbb{1} + (1-p)|G\rangle\langle G|$, then $\mathcal{W}_G^\gamma(\rho) = (1-p)(n+\gamma|E|)$. Let us define $f(\gamma) := \mathcal{W}_G^\gamma(\rho) - \gamma|E| - n + R_k^\gamma$ [the L.H.S. of Eq. (6) without the maximization], then

$$f(\gamma) = \begin{cases} \gamma(r - p|E|) - pn + 1 & \text{for } \gamma \leqslant \gamma_r^*, \\ -\gamma(p|E| - \lceil\frac{r}{2}\rceil) - pn + \lfloor\frac{r}{2}\rfloor & \text{for } \gamma \geqslant \gamma_r^*. \end{cases} \tag{S3}$$

It is easy to see that when

$$\frac{\lceil r/2\rceil}{|E|} < p < \frac{r}{|E|}, \tag{S4}$$

the function f reaches its unique maximum at γ_r^*. In order to certify r-inseparability of the state ρ, we also need $f(\gamma_r^*) > 0$, which happens when

$$p < \frac{(r+1)\lfloor\frac{r}{2}\rfloor - r}{n\lfloor\frac{r}{2}\rfloor + |E|(\lfloor\frac{r}{2}\rfloor - 1)} =: p_{\gamma_r^*}^{\max}. \tag{S5}$$

By combining Eqs. (S4) and (S5), we see that if the white-noise ratio satisfies

$$\frac{\lceil r/2\rceil}{|E|} < p < p_{\gamma_r^*}^{\max} \leqslant \frac{r}{|E|}, \tag{S6}$$

then we have found the desirable examples for Observation 1. Finally, using the fact that $n = 2(r-1)$ and $|E| = \frac{1}{2}r(r-1) + 1$ for Cthulhu graphs, we can verify that the inequalities in Eq. (S6) can hold for $r = 4$ and $r \geqslant 6$, thereby giving the final expression in Eq. (7).

Supplementary Note 4. Algorithm for calculating the criterion upper bound

This appendix provides an algorithm (Algorithm 1) for calculating $R_k^\gamma := \min_{\text{all } k\text{-cuts}}\big(\gamma|\overline{V}^{(k)}| + (1-\gamma)|\overline{E}_{\mathrm{mcm}}^{(k)}|\big)$. It uses (i) Algorithm Y from Ref. [28] (see Algorithm 2) for enumerating all unique k partitions of n items, and (ii) the blossom algorithm [29] or the Micali-Vazirani algorithm [30] for finding the maximum-cardinality matching as subroutines. In Python, one can use the function `max_weight_matching` from the NetworkX package, which implements the blossom algorithm [31]. Note that the adjacency list of a graph G is defined to be $A_G = \{1 : N(1), \dots, n : N(n)\}$ where $N(i)$ is the neighbourhood of the vertex i.

To make Algorithm 2 more intuitive, let us go through the basic ideas behind the algorithm for enumerating all k-partitions of n objects from Ref. [28]. The main idea behind the algorithm is to sequentially generate an array $\vec{a}$ that represents a unique k-partition in a non-repetitive manner. In Knuth's notation [32], each k-partition of n objects is represented by an n-dimensional array $\vec{a}$ with a_i equals to the label $\alpha \in \{0, \dots, k-1\}$ of the block to which the i-th object belongs (i.e., the i-th and the j-th objects belong to the same block if and only if $a_i = a_j$). To avoid two different arrays representing the same partition (e.g., 0011 and 1100 both represent the same bipartition), $\vec{a}$ should obey the *restricted growth* constraint [32] such that

$$a_1 = 0, \; a_{j+1} \leqslant 1 + \max(a_1, \dots, a_j) \text{ for } 1 \leqslant j < n. \tag{S7}$$

In the following algorithm (Algorithm Y from Ref. [28]), it takes a $(\leqslant k)$-partition input array $\vec{a}$ that obeys Eq. (S7) and outputs the next restricted growth array corresponding to a k-partition in the sequence. If we start with an initial array $\vec{a}_0 = [0, \dots, 0]$, by feeding the output from the previous run of the algorithm as the input to the next run, we can recursively generate all possible k-partitions of n objects. The total number of k-partitions is given by the *Stirling number of the*

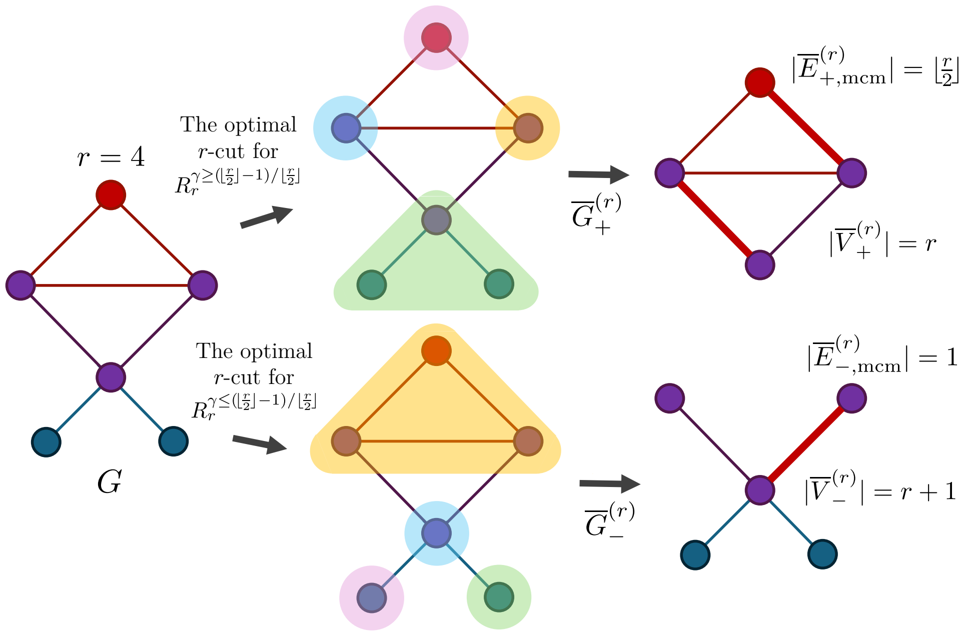

Supplementary Fig. S1. Illustration of an example corresponding to an optimal k-inseparability criterion with $\gamma \in (0, 1)$. For a Cthulhu graph G of $r = 4$ (see Fig. 2 for the meaning of r), the optimal r-cuts of $R_k^\gamma := \min_{\text{all } k\text{-cuts}}\big(\gamma|\overline{V}^{(k)}| + (1-\gamma)|\overline{E}_{\text{mcm}}^{(k)}|\big)$ for different values of $\gamma \in [0, 1]$ are shown in the middle column where the color shadings represent partitioning of the graph into r different parts. In general, for $r = 4$ and $r \geqslant 6$, the optimal r-cut for $\gamma \geqslant \big(\lfloor \frac{r}{2} \rfloor - 1\big)/\lfloor \frac{r}{2} \rfloor$ (here $= \frac{1}{2}$) results in the "head" subgraph $\overline{G}_{+}^{(r)}$, whereas the optimal r-cut for $\gamma \leqslant \big(\lfloor \frac{r}{2} \rfloor - 1\big)/\lfloor \frac{r}{2} \rfloor$ results in the "tentacles" subgraph $\overline{G}_{-}^{(r)}$. The transition of the optimal r-cut in γ from 0 to 1 also leads to a transition in R_r^γ [see Eq. (S2)].

second kind [33, §24.1.4]

$$S(n,k) = \sum_{j=0}^{k} \frac{(-1)^{k-j} j^n}{(k-j)!\,j!}, \tag{S8}$$

which should coincide with the number of arrays Algorithm 2 with input $\vec{a}_0$ can generate recursively until f_{end} hits 1.

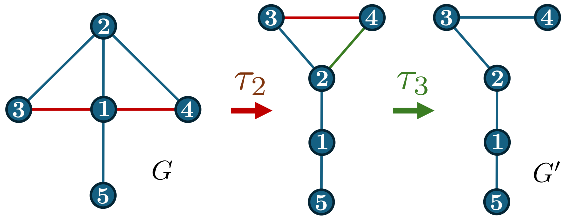

Supplementary Fig. S2. Illustration of how graph-local complementations (corresponding to local Clifford operations on graph states) can reduce the number and weight of stabilizers required by our criteria. The initial graph G has 6 edges and maximum neighboring degrees 4 and 3, so $\mathcal{W}_G^{\gamma>0}$ requires 11 stabilizers with a maximum weight of 7. To reduce both quantities, we first apply a local complementation at vertex 2, τ_2, which removes edges $(1,3)$ and $(1,4)$, and adds $(3,4)$. A subsequent local complementation at vertex 3, τ_3, removes the edge $(2,4)$. The resulting graph G' is a chain with 4 edges and maximum neighboring degrees 2 and 2, so $\mathcal{W}_{G'}^{\gamma>0}$ requires only 9 stabilizers with a reduced maximum weight of 4.

Supplementary Note 5. Reducing the number or weight of required stabilizers with local Clifford transformations

In this appendix we present an explicit example of how applying graph-local complementations, which correspond to applying local Clifford (LC) operations to graph states, can reduce the number or weight of stabilizers required by our criteria in Eq. (2). We begin by reviewing the definition of local complementation and its relation to LC transformations of graph states.

A local complementation of a graph G at vertex v, denoted $\tau_v(G)$, removes (adds) all edges that (do not) already exist between the vertices in the neighborhood of v, $N(v)$. Formally, we have [34, 35]

$$\tau_v : G \mapsto \tau_v(G) := G + N(v)\,. \tag{S9}$$

For each local complementation, there is a corresponding LC operation $U_v^\tau(G)$ that transforms the graph state $|G\rangle$ to another graph state $|\tau_v(G)\rangle$ [35]:

$$|\tau_v(G)\rangle = U_v^\tau(G)|G\rangle\,, \tag{S10}$$

$$U_v^\tau(G) := e^{-i\frac{\pi}{4}X_v} \otimes_{\alpha\in N(v)} e^{i\frac{\pi}{4}Z_\alpha} \propto \sqrt{S_v}\,, \tag{S11}$$

where S_v is the stabilizer generator of $|G\rangle$ associated with the vertex v.

Assume that we want to certify GME/k-inseparability of a state close to the graph state $|G\rangle$, which corresponds to the

Algorithm 1: Compute R_k^γ

input : n (total number of qubits/vertices in G),
k (number of non-empty blocks in the partition),
A_G (adjacency list of graph G),
$\gamma \in [0,1]$
output: $y = R_k^\gamma := \min_{\text{all } k\text{-cuts}} \big(\gamma|\overline{V}^{(k)}|+(1-\gamma)|\overline{E}^{(k)}_{\text{mcm}}|\big)$
begin
  Set $\vec{a} \leftarrow [0,\dots,0] \in \{0,\dots,k-1\}^n$
  Set $y \leftarrow n$ and $f_{\text{end}} \leftarrow 0$
  while $f_{end} = 0$ **do**
    $(\vec{a}, f_{\text{end}}) \leftarrow$ Algorithm 2 with inputs $(n,k,\vec{a})$
    Set $A_{\overline{G}^{(k)}} \leftarrow A_G$ (copy adjacency list of G)
    for i *from* 1 *to* n **do**
      for $j \in N(i)$ **do**
        if $a_i = a_j$ **then**
          Remove j from $A_{\overline{G}^{(k)}}[i]$
        end
      end
      if $|A_{\overline{G}^{(k)}}[i]| = 0$ **then**
        Remove i from $A_{\overline{G}^{(k)}}$
      end
    end
    Set $|\overline{V}^{(k)}| \leftarrow |A_{\overline{G}^{(k)}}|$ and $\overline{E}^{(k)}_{\text{mcm}} \leftarrow$ Blossom/ Micali-Vazirani algorithm with input $A_{\overline{G}^{(k)}}$
    Set $x \leftarrow \gamma|\overline{V}^{(k)}| + (1-\gamma)|\overline{E}^{(k)}_{\text{mcm}}|$
    if $x < y$ **then**
      Set $y \leftarrow x$
    end
  end
  return y
end

Algorithm 2: Enumerate all k partitions (Alg. Y [28])

input : n (total number of objects to be partitioned),
k (number of non-empty blocks in the partition),
$\vec{a}$ (input array with $a_i \leqslant k-1$ satisfying (S7))
output: $\vec{a}$ (output array representing a new k-partition)
f_{end} (output bit indicating if the algorithm has reached the end of enumerating all k-partitions)
begin
  Set $\vec{b} \leftarrow [0,\dots,0] \in \{0,\dots,k-1\}^n$
  for i *from* 2 *to* n **do**
    Set $b_i \leftarrow \max(a_{i-1}, b_{i-1})$
  end
  Set $c \leftarrow n$ and $f_{\text{end}} \leftarrow 0$
  while $a_c = k-1$ *or* $a_c > b_c$ **do**
    Set $c \leftarrow c-1$
    if $c = 1$ **then**
      Set $f_{\text{end}} \leftarrow 1$
    end
  end
  if $f_{end} = 0$ **then**
    Set $a_c \leftarrow a_c + 1$
    for j *from* $c+1$ *to* n **do**
      Set $a_j \leftarrow 0$ and $b_j \leftarrow \max(a_{j-1}, b_{j-1})$
    end
  end
  if $\max(a_n, b_n) \neq k-1$ **then**
    for r *from* 1 *to* $k-1$ **do**
      if $k - r > b_{n-r+1}$ **then**
        Set $a_{n-r+1} \leftarrow k-r$
      else
        return $\vec{a}$, f_{end}
      end
    end
  end
  return $\vec{a}$, f_{end}
end

leftmost graph in Fig. S2. Since G has 6 edges and maximum neighboring degrees 4 and 3, our criteria $\mathcal{W}_G^{\gamma>0}$ requires measuring 11 stabilizers with maximum weight 7. We can reduce both the number and maximum weight of the required stabilizers by first applying a local complementation at vertex 2, τ_2. This removes edges $(1,3)$ and $(1,4)$, and simultaneously adds $(3,4)$. We then apply a subsequent local complementation at vertex 3, τ_3, which removes the edge $(2,4)$. The resulting graph G' is a 1D chain with 4 edges and maximum neighboring degrees 2 and 2, thereby reducing the required number of stabilizers to 9 and the maximum stabilizer weight to 4. In terms of LC operations, the two graph states are related by

$$|G'\rangle = |\tau_2 \circ \tau_3(G)\rangle = U_3^\tau(\tau_2(G))U_2^\tau(G)|G\rangle\,. \tag{S12}$$

As mentioned after Remark 1 in Sec. II B, we can define a new criterion $\widetilde{\mathcal{W}}_{G'}^{\gamma}$ for certifying GME/k-inseparability of states close to $|G\rangle$ by replacing the stabilizers of $|G'\rangle$ in Eq. (2) with their LC-conjugated counterparts:

$$\begin{aligned}\widetilde{\mathcal{W}}_{G'}^{\gamma}(\rho) &= \sum_{i\in V'} |\langle \widetilde{U}^\dagger S_i \widetilde{U}\rangle_\rho| + \gamma \sum_{(i,j)\in E'} |\langle \widetilde{U}^\dagger S_i S_j \widetilde{U}\rangle_\rho|,\\ \widetilde{U} &:= U_3^\tau(\tau_2(G))U_2^\tau(G)\,,\end{aligned} \tag{S13}$$

so that $\widetilde{U}^\dagger S_i \widetilde{U}$ for $i\in V'$ and $\widetilde{U}^\dagger S_i S_j \widetilde{U}$ for $(i,j)\in E'$ are all stabilizers of the original graph state $|G\rangle$. Since conjugating any local operator, such as a stabilizer, with local unitaries cannot increase its weight, the maximum weight of the 9 LC-conjugated stabilizers appearing in Eq. (S13) remains 4.

Supplementary Note 6. A brief review of SDP

The purpose of this appendix is to briefly review the key concepts and properties of SDP that we use in the main text. The objective of SDP is to minimize or maximize a linear function with respect to a positive semi-definite matrix subject to some linear equality or inequality constraints. The standard form of an SDP (primal) problem takes the following form [36, 37]:

$$\alpha := \min_{\tilde{X}} \langle A, \tilde{X}\rangle \tag{S14a}$$

$$\text{subject to } \langle B_i, \tilde{X}\rangle = b_i \text{ for } i = 1,\dots,r, \tag{S14b}$$

$$\langle C_j, \tilde{X}\rangle \leqslant c_j \text{ for } j = 1,\dots,s, \tag{S14c}$$

$$\tilde{X} \geqslant 0, \tag{S14d}$$

where A, B_i, C_j are Hermitian matrices and $\vec{b}, \vec{c}$ are real vec-

tors. The associated dual problem is given by

$$\beta := \max_{\vec{y},\vec{z}} \ \vec{b}^T\vec{y} + \vec{c}^T\vec{z} \tag{S15a}$$

$$\text{subject to } \sum_{i=1}^{r} y_i B_i + \sum_{j=1}^{s} z_j C_j \leqslant A, \tag{S15b}$$

$$\vec{y} \in \mathbb{R}^r, \ \vec{z} \leqslant \vec{0}_s, \tag{S15c}$$

where $\langle \tilde{X}, \tilde{Y}\rangle = \mathrm{Tr}(\tilde{X}^\dagger \tilde{Y})$ is the Hilbert-Schmidt inner product between two matrices $\tilde{X}$ and $\tilde{Y}$. Note that the above definitions are not in the exact forms as in Refs. [36, 37], but all these definitions are equivalent.

By *weak duality* of SDP [37], the optimal solution to the primal problem α is always lower bounded by the optimal solution to the dual problem β (i.e., $\alpha \geqslant \beta$). This means that any feasible solution to the dual problem will be a reliable lower bound for the true optimal solution to the primal problem. Therefore, suboptimal solutions to the dual problem provided by any numerical solver will in principle not overestimate the true value of α.

Furthermore, if the primal/dual feasible set satisfies additional conditions, then *strong duality* holds, in which case the optimal primal solution equals to the optimal dual solution (i.e., $\alpha = \beta$). These conditions are called *Slater's condition* which is stated in the following lemma.

Lemma 3 (Slater's theorem for SDP [37]). *The following two statements hold for all SDPs:*

1. *If α is finite (i.e., the primal feasible set is non-empty) and there exist vectors $\vec{y} \in \mathbb{R}^r$ and $\vec{z} \in \mathbb{R}^s$ which satisfy strict inequality for all constraints in the dual problem (i.e., $\exists\ \vec{y} \in \mathbb{R}^r$ and $\vec{z} < \vec{0}_s$ such that $\sum_{i=1}^{r} y_i B_i + \sum_{j=1}^{s} z_j C_j < A$), then $\alpha = \beta$. Also, there exists a feasible $\tilde{X}$ such that $\langle A, \tilde{X}\rangle = \alpha$.*

2. *If β is finite (i.e., the dual feasible set is non-empty) and there exist a positive definite matrix $\tilde{X} > 0$ which satisfies all equality constraints $\langle B_i, \tilde{X}\rangle = b_i$ and all inequality constraints with strict inequality (i.e., $\langle C_j, \tilde{X}\rangle < c_j$) of the primal problem, then $\alpha = \beta$. Also, there exist a feasible pair $\vec{y}, \vec{z}$ such that $\vec{b}^T\vec{y} + \vec{c}^T\vec{z} = \beta$.*

It is sufficient to prove strong duality by showing either the primal or dual problem satisfies one of the above Slater's conditions. If the Slater's condition is satisfied, strong duality will ensure the best numerical solution (potentially suboptimal) to the dual problem obtained by numerical solvers to be a tight lower bound to α.

Supplementary Note 7. Theoretical examples

In order to measure all the terms of our GME and k-inseparability criteria in Eq. (2) corresponding to any connected graphs with at least four vertices, one must be able to measure at least up to 4-body correlators. In this section, we will investigate the noise tolerance of our criteria for the states $\rho_G(p) = \frac{p}{2^n}\mathbb{1} + (1-p)|G\rangle\langle G|$, and later also for non-stabilizer states that are LU equivalent to (noisy) Dicke states. By applying Eq. (2) to $\rho_G(p)$, we have that

$$\mathcal{W}_G^\gamma(\rho_G(p)) = (1-p)(n + \gamma|E|). \tag{S16}$$

In order to certify that $\rho_G(p)$ is k-inseparable, the white-noise ratio must satisfy

$$p < \max_{0\leqslant\gamma\leqslant 1} \frac{R_k^\gamma}{n+\gamma|E|} =: p_k^{\max} \tag{S17}$$

with $R_k^\gamma = \min_{\text{all } k\text{-cuts}}\big(\gamma|\overline{V}^{(k)}| + (1-\gamma)|\overline{E}^{(k)}_{\text{mcm}}|\big)$ by Theorem 1. Given that the maximum number of qubits need to be simultaneously measured when applying our GME/k-inseparability criteria grows linearly with the maximum degree of the graph, we will only focus on examples associated to graphs with constant maximum degree (i.e., the maximum degree of the graph does not grow with the number of vertices n) in the following (and also the star graphs and complete graphs). This is well motivated by the measurement restriction of the experimental settings that we consider in Sec. II D which we are only allowed to measure $\leqslant O(1)$-body observables.

In fact, some of these graphs are associated to graph states that have important applications in quantum information processing. For example, ring-graph states are the building blocks for fusion-based quantum computing [38]. Furthermore, cluster states—corresponding to 2D lattices—are the essential ingredients for MBQC [39]. Tree-graph states also have applications in error correction for MBQC [40] and in constructing one-way quantum repeaters [41]. Therefore, certifying multipartite entanglement of these states is also motivated from a practical perspective.

a. Graphs requiring $\leqslant$4-body correlators in $\mathcal{W}_G^\gamma$

Assuming that we can measure up to 4-body correlators, there are a limited sets of graph states of which all the stabilizer terms in our GME and k-inseparability criteria in Eq. (2) can be measured. The full list of the associated graphs consists of all 3-vertex, 4-vertex graphs, and all n-vertex path/chain graphs and ring graphs with $n \geqslant 5$. Note that the only two connected 3-vertex graphs are the chain graph and ring graph.

We first consider states $\rho_G(p)$ associated to connected graphs that are neither a complete graph, a star graph, a chain graph nor a ring graph, which are the 4-qubit states that correspond to the graphs in Fig. S3. Using Algorithms 1 and 2, we find that for all $2 \leqslant k \leqslant 4$, $R_k^\gamma = \gamma k + (1-\gamma)\lfloor \frac{k}{2}\rfloor$ and $p_k^{\max} = \frac{k}{8}$ for graph (a); $R_k^\gamma = \gamma \min(k+1, 4) + (1-\gamma)\lceil \frac{k}{2}\rceil$ and $p_k^{\max} = \frac{1}{3}$ (for $k=2$) and $\frac{1}{2}$ (for $k = 3, 4$) for both graphs (b) and (c). For 4-qubit star graph and complete graph states, please refer to Supplementary Note 7 c.

Next, we consider states $\rho_G(p)$ associated to the n-vertex chain/path graphs [see Fig. S4 (a)]. These states are also known as *1D cluster* states. By running Algorithms 1 and 2, we verified for $3 \leqslant n \leqslant 12$ that $R_k^\gamma = \gamma k + (1-\gamma)\lfloor \frac{k}{2}\rfloor$ and $p_k^{\max} = \frac{k}{2n-1}$ (optimal γ: $\gamma^* = 1$) for all $2 \leqslant k \leqslant n$.

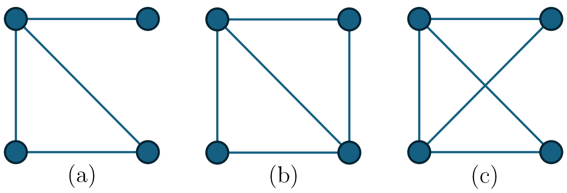

Supplementary Fig. S3. List of all non-isomorphic connected 4-vertex graphs excluding the complete, star, chain/path, and ring graphs.

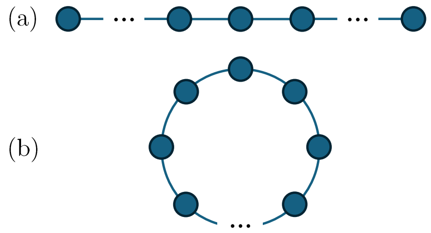

Supplementary Fig. S4. The n-vertex (a) path graph and (b) ring graph.

Similarly, for n-vertex ring graphs [see Fig. S4 (b)], we verified for $3 \leqslant n \leqslant 12$ that $R_k^\gamma = \gamma \min(k+1, n) + (1-\gamma)\min(\lceil \frac{k}{2} \rceil, \lfloor \frac{n}{2} \rfloor)$ and $p_k^{\max} = \frac{\min(k+1,n)}{2n}$ ($\gamma^* = 1$) for all $2 \leqslant k \leqslant n$. Note that to evaluate the function $\mathcal{W}_G^\gamma$ associated to any chain graphs or ring graphs only require at most 4-body stabilizer expectation values independent of the number of vertices/qubits n.

For graphs with more vertices (i.e., $n > 12$), calculating the reduction term R_k^γ can become too computationally costly. In those cases, we can apply the looser bound in the second inequality of Eq. (4), which gives an easily computable GME and k-inseparability criterion

$$\max_{0\leqslant\gamma\leqslant1} \mathcal{W}_G^\gamma(\rho) - \gamma(|E| - k + 1) - n + 1 > 0. \tag{S18}$$

Using this relaxed criterion, we can still certify k-inseparability for larger graph states with white noise

$$p < \max_{0\leqslant\gamma\leqslant1} \frac{1+\gamma(k-1)}{n+\gamma|E|} =: p_k^{\text{max, loose}}. \tag{S19}$$

In particular, for general path graphs with $|E| = n-1$, we can apply these relaxed criteria to obtain (looser) bounds for the k-inseparability white-noise thresholds $p_k^{\text{max, loose}} = \frac{k}{2n-1}$ ($\gamma^* = 1$) for all $2 \leqslant k \leqslant n$ and $n \geqslant 2$, coinciding with the supposedly tighter threshold proven for $n \leqslant 12$ above. As for general ring graphs with $|E| = n$, the (looser) bound for the threshold is $p_k^{\text{max, loose}} = \frac{k}{2n}$ ($\gamma^* = 1$) for all $2 \leqslant k \leqslant n$ and $n \geqslant 2$.

b. Graphs requiring $4 < m \leqslant O(1)$-body correlators in $\mathcal{W}_G^\gamma$

In the following, we will focus on graphs with constant maximum degree. These include all 2D lattices with each vertex having at most 4 neighbors and all tree graphs with the maximum degree $\leqslant O(1)$ (see Fig. S5).

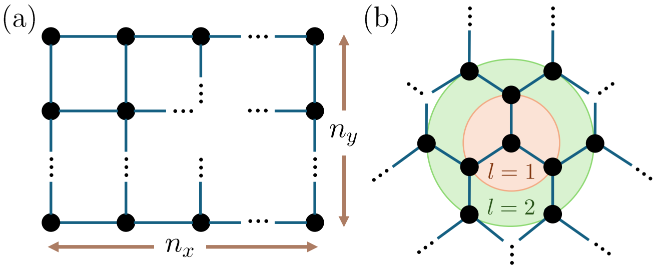

Supplementary Fig. S5. Examples of graphs associated with $4 < m \leqslant O(1)$-body correlators in $\mathcal{W}_G^\gamma$. (a) A 2D lattice with n_x and n_y labeling the number of vertices in each direction. (b) A degree-3 tree graph with depth $D \geqslant 2$ where l labels the level of branches from the root node.

Since the number of k-partitions grows as $O(k^n)$, we only verified the following white-noise tolerance relationships with Algorithms 1 and 2 for a limited number of (n_x, n_y) associated to 2D lattices [see Fig. S5 (a)]. For $2 \leqslant n_x \leqslant 6$, $n_y = 2$, and $2 \leqslant k \leqslant 2n_x$, we verified that $R_k^\gamma = \gamma \min(k+1, 2n_x) + (1-\gamma)\min(\lceil \frac{k}{2} \rceil, n_x)$ and since $n = 2n_x$ and $|E| = 3n_x - 2$, the corresponding maximum white-noise ratio for violating the k-separability condition in Theorem 1 is given by

$$p_k^{\max} = \begin{cases} \frac{3}{5n_x-2} & \text{for } k=2 \ (\gamma^*{=}1), \\ \frac{1}{n_x} & \text{for } k=3, \text{and if } n_x=2, \text{also for } k=4 \ (\gamma^*{=}0), \\ \frac{5}{5n_x-2} & \text{for } k=4 \text{ if } n_x \geqslant 3 \ (\gamma^*{=}1), \\ \frac{\lceil k/2 \rceil}{2n_x} & \text{for all } 5 \leqslant k \leqslant 2n_x \text{ if } n_x \geqslant 3 \ (\gamma^*{=}0). \end{cases} \tag{S20}$$

For $3 \leqslant n_x \leqslant 4, n_y = 3$, and $2 \leqslant k \leqslant 3n_x$, we verified that $R_k^\gamma = \gamma \min(k+a+1, 3n_x) + (1-\gamma)\min(\lceil \frac{k}{2} \rceil, \lfloor \frac{3n_x}{2} \rfloor)$ where

$$a = \begin{cases} 1, & \text{if } 3 \leqslant k \leqslant 3(n_x-1), \\ 0, & \text{else}, \end{cases} \tag{S21}$$

and since $n = 3n_x$ and $|E| = 5n_x - 3$, we obtain

$$p_k^{\max} = \begin{cases} \frac{k+a+1}{8n_x-3} & \text{for } k \in \{2,3,4\} \cup \mathcal{I}_{\text{even}}, \\ \frac{\min(\lceil \frac{k}{2} \rceil, \lfloor \frac{3n_x}{2} \rfloor)}{3n_x} & \text{for } k \in \{3n_x-2 \leqslant m \leqslant 3n_x\} \cup \mathcal{I}_{\text{odd}}, \end{cases} \tag{S22}$$

where $\mathcal{I}_{\text{odd/even}} := \{m \in \mathbb{N}_{\text{odd/even}} | 5 \leqslant m \leqslant 3(n_x-1)\}$, and the optimality in the first and second cases is attained for the optimal choices $\gamma^* = 1$ and $\gamma^* = 0$, respectively.

To bound the white-noise threshold for cluster states with more than 12 qubits, we apply the relaxed criteria in Eqs. (S18) and (S19). For general cluster states where $n = n_x \times n_y$ and $|E| = 2n - n_x - n_y$, we certify k-inseparability if the white-noise ratio is below the looser threshold bound

$$p_k^{\text{max, loose}} = \begin{cases} \frac{1}{n} & \text{for } k=2 \ (\gamma^*{=}0), \\ \frac{k}{3n-n_x-n_y} & \text{for } k \geqslant 3 \ (\gamma^*{=}1). \end{cases} \tag{S23}$$

Moving on to another class of graph states for which our GME/k-inseparability criteria require at most $O(1)$-body measured observables, we now consider tree graphs with constant maximum degree. For illustration, we focus on degree-3 tree graphs. In Fig. S5 (b), the integer l denotes the level of branches extending from the root vertex and the graph's depth D is equal to the maximum level. In general, the number of vertices and edges in a depth-D degree-3 tree graph are $n = 3(2^D - 1) + 1$ and $|E| = n - 1$. With Algorithms 1 and 2, the bound reduction term is found to be $R_k^\gamma = k\gamma + (1-\gamma)\lceil \frac{k-1}{3} \rceil$ for $D = 1, 2$ (i.e., $n = 4, 10$). Hence, the corresponding white-noise thresholds for certifying k-inseparability are

$$p_k^{\max} = \frac{k}{2n-1} \quad \text{for all } 2 \leqslant k \leqslant n \ (\gamma^* = 1). \tag{S24}$$

For larger depths $D \geqslant 3$ (corresponding to $n \geqslant 22$), it will take too long to compute R_k^γ. Therefore, we can use the looser bound in Eqs. (S18) and (S19) instead, which coincidentally lead to the same white-noise thresholds appearing in Eq. (S24). In fact, since all tree graphs (with any degree and depth) have $|E| = n - 1$ number of edges, the white-noise thresholds appearing in Eq. (S24) hold for all tree graphs.

c. *Star graphs and complete graphs*

To complement our investigation of graph states, we also consider star-graph and complete-graph states. Since these two types of graphs have maximum degree n, any existing witness/criterion that certifies GME/k-inseparability of these states (including ours) involve measuring n-body stabilizers. In fact, it has been proven that any method that can certify GME of these n-qubit states must measure at least one n-body observable [42]. For n-vertex star graphs, we verified with Algorithms 1 and 2 for $3 \leqslant n \leqslant 12$ that $R_k^\gamma = \gamma(k-1) + 1$, so the maximum white-noise tolerance for violating the k-separability condition is $p_k^{\max} = \frac{k}{2n-1}$ $(\gamma^* = 1)$ for all $2 \leqslant k \leqslant n$. For n-vertex complete graphs, we also verified that $R_k^\gamma = \gamma n - (1-\gamma)\min(k-1, \lfloor \frac{n}{2} \rfloor)$ for $3 \leqslant n \leqslant 12$, so

$$p_k^{\max} = \begin{cases} \frac{2}{n+1} \text{ for } k = 2, \text{ and if } n = 3, \text{also for } k = 3, \\ \frac{\min(k-1, \lfloor n/2 \rfloor)}{n} \text{ for all } 3 \leqslant k \leqslant n \text{ if } n \geqslant 4, \end{cases} \tag{S25}$$

where the optimality in the first and second cases is attained for the optimal choices $\gamma^* = 1$ and $\gamma^* = 0$, respectively.

We remark that based on some empirical observations with star-graph states, the SDP method from Sec. II C has not been able to provide non-trivial lower bounds for the absolute expectation values of any n-body stabilizer that appears in Eq. (2) using only $O(1)$-body Pauli expectation values as constraints. This leads us to believe that our SDP technique for bounding unmeasured stabilizer terms works only when all the stabilizer generators are measured.

d. *Noisy Dicke states*

Finally, to demonstrate the general applicability of our criteria, let us show that our criteria can also detect GME in non-stabilizer states. More specifically, we consider states $\rho_{D(n,i)}(p, \vec{\theta})$ that are LU equivalent to (noisy) Dicke states, which are particularly relevant in quantum many-body physics [43, 44] and have broad applications in quantum information processing [45, 46]. These states are defined as

$$|D_n^{(i)}\rangle = \binom{n}{i}^{-\frac{1}{2}} \sum_{x \in \{0,1\}^n : \mathrm{wt}(x) = i} |x\rangle, \tag{S26}$$

$$\rho_{D(n,i)}(p, \vec{\theta}) = \frac{p}{2^n}\mathbb{1} + (1-p) U_{\vec{\theta}} |D_n^{(i)}\rangle\langle D_n^{(i)}| U_{\vec{\theta}}^\dagger, \tag{S27}$$

where $U_{\vec{\theta}} = \bigotimes_{i=1}^n R_z(\theta_{3i-2}) R_y(\theta_{3i-1}) R_z(\theta_{3i})$ with $\vec{\theta} \in [-\pi, \pi]^{3n}$, $R_z(\varphi) = e^{-i\varphi Z/2}$, and $R_y(\varphi) = e^{-i\varphi Y/2}$. Using our criteria defined for the complete graph with $\gamma = 1$, we can certify GME in $\rho_{D(n,i)}(p, \vec{\theta})$ for various parameters $n, i, \vec{\theta}$ if the white-noise ratio satisfies $p < p_c$ as shown in Table S1.

In summary, we have shown that our GME/k-inseparability criteria can tolerate a wide range of white noise in various families of graph states with important applications in quantum information, as well as in non-stabilizer states such as Dicke states (up to LU), which are relevant in quantum many-body physics.

Supplementary Note 8. Measuring Pauli observables of microwave photons

In this appendix, let us briefly review how Pauli observables for microwave photons can be measured using heterodyne measurement, following Ref. [47, 48]. This measurement scheme also motivates the restriction of the maximum weight of the Pauli observables that can be reliably measured in a realistic measurement setup.

In heterodyne detection, the complex integrated signal of a single photonic mode corresponds to a noisy observable $\hat{S} = \hat{a} + \hat{h}^\dagger$, where $\hat{a}$ is the annihilation operator acting on the photonic mode, and $\hat{h}$ acts on the noise field, which in the ideal case corresponds to a vacuum mode, but in state-of-the-art experiments, is typically in a thermal state with 2-4 noise photons. Averaging over experimental repetitions allows measuring multi-mode moments

$$\left\langle \prod_i^N (S_i^\dagger)^{s_i} (S_i)^{t_i} \right\rangle = \sum_{p_1, q_1, \ldots p_N, q_N = 0}^{s_1, t_1, \ldots, s_N, t_N} \left[\prod_i^N \binom{s_i}{p_i} \binom{t_i}{q_i} \right] \times \left\langle \prod_i^N (a^\dagger)^{p_i} a^{q_i} \right\rangle \left\langle \prod_i^N h^{s_i - p_i} (h^\dagger)^{t_i - q_i} \right\rangle, \tag{S28}$$

where i is the index for the photonic mode and n is the total number of photonic modes. By measuring the noise moments independently, one can find the signal moments by inverting

n	i	θ_1	θ_2	θ_3	θ_4	θ_5	θ_6	θ_7	θ_8	θ_9	θ_{10}	θ_{11}	θ_{12}	θ_{13}	θ_{14}	θ_{15}	θ_{16}	θ_{17}	θ_{18}	θ_{19}	θ_{20}	θ_{21}	p_c
3	2	0	-0.53	-0.33	π	-0.53	-0.33	0	-0.53	-0.33	/	/	/	/	/	/	/	/	/	/	/	/	0.315
4	2	0.82	-π	-0.23	0	-2.50	-1.05	-π	0.52	-1.05	0	-2.73	-1.05	/	/	/	/	/	/	/	/	/	0.143
5	1	-π	-0.37	1.38	-π	2.77	1.38	-π	2.77	1.38	0	-0.37	1.38	0	-0.37	1.38	/	/	/	/	/	/	0.008
5	3	-π	-0.33	1.08	π	-2.82	-2.06	π	-0.33	1.08	0	0.33	-2.06	π	2.82	1.08	/	/	/	/	/	/	0.264
6	3	0	-0.27	1.36	0	2.87	1.36	3.14	-0.27	1.36	-π	-2.87	-1.78	0	0.27	-1.78	0	-0.27	1.36	/	/	/	0.315
6	4	0	0.28	2.71	0	-2.86	2.71	0	0.28	2.71	0	-0.28	-0.43	0	0.28	2.71	-π	-0.28	-0.43	/	/	/	0.247
7	3	-π	0.28	0	0	-0.28	-3.14	3.14	2.87	π	0	-2.87	0	3.14	0.28	0	-π	2.87	-3.14	1.51	π	1.51	0.399
7	5	-π	-2.89	-2.13	0	-0.25	1.01	0	-0.25	1.01	0	2.89	1.01	0	-0.25	1.01	0	-0.25	1.01	0	2.89	1.01	0.303

Supplementary Table S1. Local rotation angles $\{\theta_j\}$ (rounded to two decimal places) defining LU-conjugated (noisy) Dicke states $|D_n^{(i)}\rangle$ for which our criteria certify GME. The last column shows the critical white-noise tolerance p_c (rounded to three decimal places).

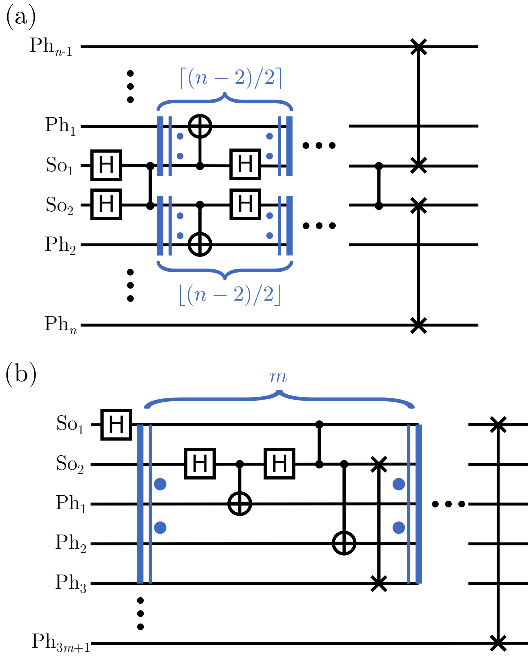

Supplementary Fig. S6. Quantum circuit for generating the ring-graph and tree-graph states. (a) Quantum circuit for generating the n-qubit ring-graph state using two source transmons. (b) Quantum circuit for generating the $(m, 2)$ tree-graph state using two source transmons.

the equation above, provided that the noise is uncorrelated with the signal. The difficulty of characterizing a particular moment is related to its *order*, given by $O = \lceil \sum_i (p_i + q_i)/2 \rceil$. The signal-to-noise ratio of measuring a moment of order O scales as η^O [49], where $\eta = 1/\langle \hat{h}\hat{h}^\dagger \rangle$ is the single-photon measurement efficiency, typically between 0.2 and 0.4 in state-of-the-art microwave-photon measurements. This means that the number of measurement repetitions required to obtain an accurate estimate of the moment scales exponentially with the order of the moments. For example, the experiment in Ref. [2] took around 10 billion repetitions, which allowed measuring order-4 moments reliably.

Assuming each photonic mode has no more than one photon, the Pauli observables can be related to the moments via

$$\hat{X} = a^\dagger + a, \tag{S29a}$$

$$\hat{Y} = i(a^\dagger - a), \text{ and} \tag{S29b}$$

$$\hat{Z} = I - 2a^\dagger a. \tag{S29c}$$

Therefore, there is a linear transformation that relates the Pauli observables to the moments. As with the moments measurements, the number of measurement repetitions required for estimating Pauli observables also scales exponentially with the weight of the observable, making high-weight Pauli observables difficult to estimate. Note that in this particular setup, the measurement difficulty is not determined by which subset of modes/qubits is measured, but rather by the number of modes/qubits being measured.

Supplementary Note 9. Simulating graph-state generation

In this section, we briefly review the protocol for generating graph states with superconducting circuits and introduce the numerical simulation procedure. We consider systems in which coherently controllable source modes are tunably coupled to a waveguide that carries itinerant photons. In superconducting circuits, the source mode can be realized by a transmon qubit. The tunable coupling to the waveguide is achieved by first tunably coupling the source transmon to an ancillary mode, which is in turn strongly coupled to the waveguide. By controlling the interaction between the source transmon's second excited state and the ancillary mode, itinerant photons can be created conditioned on the initial state of the source mode. This effectively realizes a CNOT gate between the source mode and the microwave photonic qubit, where the logical states are the vacuum and the single-photon Fock state of a particular mode. Combined with single-qubit gates on the source qubit, a pair of such source and ancillary modes allows deterministic generation of one-dimensional graph states. Extending this scheme by tunably coupling multiple source-ancillary-mode pairs makes it possible to generate ring-graph, tree-graph, and both one- and two-dimensional cluster states. The quantum circuit for generating 2D cluster states is shown in Fig. 3, while those for generating tree-graph and ring-graph states are given in Fig. S6.

To numerically simulate the graph-state generation, we implement a noisy circuit simulation in *Cirq*, following Ref. [2].

Leakage		Coherence times		Gate times	
L_{CNOT}	0.01	$T_{1,2}^{\mathrm{So}_1,\mathrm{g-e}}$	27, 22 μs	Single-qubit	125 ns
L_{CZ}	0.02	$T_{1,2}^{\mathrm{So}_2,\mathrm{g-e}}$	22, 23 μs	CZ	200 ns
		$T_{1,2}^{\mathrm{So}_1,\mathrm{e-f}}$	16, 12μs	CNOT	325 ns
		$T_{1,2}^{\mathrm{So}_2,\mathrm{e-f}}$	4, 6μs	SWAP	350 ns

Supplementary Table S2. Table of parameters used for the noisy circuit simulation. Note that we assume the leakage error and gate times are the same for both transmons. For the generation of the 3×3 cluster state, the third transmon is assumed to have the same coherence times as So_1.

The code used for our simulations is available in Ref. [50]. The source mode transmons are modeled as qutrits, with the lowest three levels g, e and f respectively, and the photons are modeled as qubits. Most of the transmon gates are performed in the computational subspace g, e, with the exception of the controlled emission and CZ gate. The controlled emission (CNOT) is modeled as a π_{e-f} gate on the transmon, followed by a swap between the $e-f$ excitation manifold and the photonic mode. The CZ gate is modeled by driving the ee state of the coupled transmon through a 2π rotation via the fg state, hence accumulating a geometric phase of -1 on the ee state.

Errors in the circuit can have both coherent and incoherent contributions. In Ref. [2], the coherent error is dominated by leakage process during the CZ and CNOT gates, meaning that the system retains some population in the f state after the gate. This can be caused by imperfect calibration of the rotation angle. Incoherent errors occur as the excited levels of the transmon qutrits have finite relaxation and dephasing times, compared to the time taken for the emission. The relaxation and dephasing processes are modeled as three-level amplitude damping and dephasing channels, respectively, applied symmetrically during the action of each gate. The damping and dephasing rates are determined based on the individual gate times, as well as the relaxation and dephasing times T_1, T_2 between the $g-e$ and $e-f$ levels, respectively. In Table S2, we summarize the simulation parameters, which were based on Ref. [2].

Supplementary Note 10. Error analysis of certifying GME/k-inseparability

In order to certify GME/k-inseparability of a state ρ prepared in any experiment, it is inevitable to address the statistical uncertainty of the measured expectation values of the stabilizers in Eq. (2). In practice, we want to show by how many standard deviations (SDs) the estimated value of $\mathcal{W}_G^\gamma(\rho)$ exceeds the k-separability bound in Theorem 1. For that, we denote one SD of a random variable $x\in[-1,1]$ corresponding to a Hermitian observable $\tilde{X}\in\mathrm{Stab}(|G\rangle)$ as $\sigma_x=\sqrt{\langle(\tilde{X}-\langle\tilde{X}\rangle_\rho)^2\rangle_\rho}$ with the mean $\mu(x)=\langle\tilde{X}\rangle_\rho$. In general, we can have correlated variables x and y (corresponding to a Hermitian observable $\tilde{Y}$) of which $\sigma_{xy}=\langle(\tilde{X}-\langle\tilde{X}\rangle_\rho)(\tilde{Y}-\langle\tilde{Y}\rangle_\rho)\rangle_\rho$. Using the standard formula for first-order uncertainty propagation under the assumption that all the stabilizer expectation values that appear in $\mathcal{W}_G^\gamma(\rho)$ are obtained from actual measurements (but not from SDPs in Sec. II C) and the variance of each variable is small enough (by performing enough measurements), we obtain the uncertainty for the function $\mathcal{W}_G^\gamma$ that appears in our GME and k-inseparability criteria

$$\sigma_{\mathcal{W}_G^\gamma}=\sqrt{\sum_{i,j\in V}\frac{\partial\mathcal{W}_G^\gamma}{\partial s_i}\frac{\partial\mathcal{W}_G^\gamma}{\partial s_j}\sigma_{s_is_j}+\sum_{\alpha\in V,\,(i,j)\in E}\frac{\partial\mathcal{W}_G^\gamma}{\partial s_\alpha}\frac{\partial\mathcal{W}_G^\gamma}{\partial s_{(i,j)}}\sigma_{s_\alpha s_{(i,j)}}+\sum_{(i,j),(\alpha,\beta)\in E}\frac{\partial\mathcal{W}_G^\gamma}{\partial s_{(i,j)}}\frac{\partial\mathcal{W}_G^\gamma}{\partial s_{(\alpha,\beta)}}\sigma_{s_{(i,j)}s_{(\alpha,\beta)}}}$$
$$=\sqrt{\sum_{i,j\in V}\frac{\langle S_i\rangle\langle S_j\rangle}{|\langle S_i\rangle||\langle S_j\rangle|}\sigma_{s_is_j}+\gamma\sum_{\alpha\in V,\,(i,j)\in E}\frac{\langle S_\alpha\rangle\langle S_iS_j\rangle}{|\langle S_\alpha\rangle||\langle S_iS_j\rangle|}\sigma_{s_\alpha s_{(i,j)}}+\gamma^2\sum_{(i,j),(\alpha,\beta)\in E}\frac{\langle S_\alpha S_\beta\rangle\langle S_iS_j\rangle}{|\langle S_\alpha S_\beta\rangle||\langle S_iS_j\rangle|}\sigma_{s_{(i,j)}s_{(\alpha,\beta)}}}, \quad (S30)$$

where the random variable $s_{(i,j)}$ is associated to the stabilizer S_iS_j and all partial derivatives are evaluated at the mean of each variable. The last equality follows from the formula $\frac{d}{dx}|x|=\frac{x}{|x|}$ for all $x\neq 0$ and holds as long as all the corresponding expectation values $\langle S_i\rangle$ and $\langle S_iS_j\rangle$ are non-zero. In any realistic scenarios, no expectation values will be exactly zero up to infinite precision, so the last equality in Eq. (S30) should apply to general experiments.

However, if we have to apply the SDP techniques in Sec. II C to lower bound the absolute expectation values of the unmeasured stabilizers in $\mathcal{W}_G^\gamma(\rho)$, one will need to find the partial derivatives of the optimal solution of each associated SDP problem with respect to each constraint parameter that corresponds to a measured variable in order to evaluate $\sigma_{\mathcal{W}_G^\gamma}$. The exact calculations go beyond the scope of this paper, for which the relevant technique is described in Ch. 5.3.6 of Ref. [51]. Alternatively, one can estimate the partial derivatives of $\mathcal{W}_G^\gamma(\rho)$ by taking the partial derivative of the dual objective function in Eq. (23a) with respect to the expectation value b_j for the measured observable B_j as part of the SDP constraint. For example, if the term $|\langle S_iS_j\rangle_\rho|$ is lower bounded by an SDP described in Sec. II C [i.e., having $|\langle S_iS_j\rangle_\rho|$ replaced by $\beta(|\langle S_iS_j\rangle_\rho|)$ in $\mathcal{W}_G^\gamma(\rho)$], which has a set of constraints: $|\mathrm{Tr}(B_m\rho)-b_m|\leqslant\varepsilon_m$ with $\varepsilon_m=\sigma_{b_m}$ labelled by m [see Eq. (21b)], then we get an estimate $\frac{\partial\beta(|\langle S_iS_j\rangle_\rho|)}{\partial b_m}=y^*_{2m-1}-y^*_{2m}$ with $\vec{y}^*$ being part of the op-

timal solution of the dual SDP problem in Eqs. (23a)-(23d). The overall estimate of $\sigma_{\mathcal{W}_G^\gamma}$ will take a similar form as in Eq. (S30) but now having terms that depend on $\frac{\partial\beta(|\langle S_iS_j\rangle_\rho|)}{\partial b_m}$, $\sigma_{s_\alpha b_m}$ and $\sigma_{b_m b_{m'}}$ instead of $\frac{\partial \mathcal{W}_G^\gamma}{\partial s_{(i,j)}}$, $\sigma_{s_\alpha s_{(i,j)}}$ and $\sigma_{s_{(\alpha,\beta)} s_{(i,j)}}$.

Supplementary Note 11. More simulation results

In this final section, we present the results of the GME/k-inseparability certification for 1D and 2D cluster states as well as tree-graph states, see Tables S3, S4, and S5, respectively. The corresponding state generation has been simulated under realistic experimental conditions (see Secs. II D, II E, and Supplementary Note 9). Although the GME/k-inseparability witnesses of Ref. [7] outperform our criteria, their method generally requires measuring at least $O(2^{n/c})$ stabilizers, with maximum weight scaling as $O(n)$, where c is the chromatic number of the underlying graph. Such measurement requirement is beyond the experimental capabilities of the platforms considered here for large n.

Supplementary Table S3: 1D cluster states

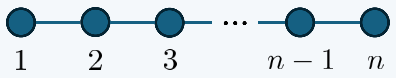

n	[4]'s witness	[7]'s witness	Our criteria	Our criteria (SDP)	Fidelity
4	GME	GME	GME	GME	0.888
5	GME	GME	GME	GME	0.855
6	GME	GME	GME	GME	0.823
7	GME	GME	GME	GME	0.793
8	/	GME	3-insep	4-insep	0.763
9	/	GME	3-insep	4-insep	0.735
10	/	GME	3-insep	4-insep	0.708
11	/	4-insep	4-insep	4-insep	0.683
12	/	4-insep	4-insep	4-insep	0.660

Comparison of certified multipartite entanglement in simulated n-qubit 1D cluster states using different witnesses/criteria. The first column shows the GME certification results using the witness from Eq. (45) in Ref. [4]. The second column shows certification results using the witness in Eq. (21) of Ref. [7]. The third column reports GME/k-inseparability certified by our criteria with all terms in Eq. (2) measured. The fourth column shows results from our criteria with only the stabilizer generators measured, and all $|\langle S_iS_j\rangle|$ in Eq. (2) lower bounded by the dual SDP in Sec. II C. The last column gives the fidelities with the ideal graph states, calculated from the full simulated density matrices.

Supplementary Table S4: 2D cluster states

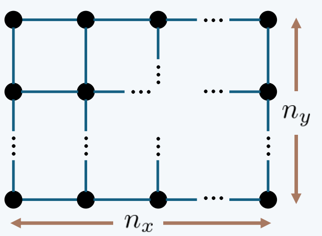

n_x	n_y	[4]'s witness	[7]'s witness	Our criteria	Our criteria (SDP)	Fidelity
2	2	GME	GME	GME	GME	0.838
3	2	/	GME	3-insep	3-insep	0.748
4	2	/	3-insep	3-insep	3-insep	0.662
5	2	/	3-insep	5-insep	5-insep	0.587
6	2	/	5-insep	7-insep	7-insep	0.521
3	3	/	3-insep	5-insep	5-insep	0.615

Comparison of certified multipartite entanglement in simulated $n_x \times n_y$-qubit 2D cluster states using different witnesses/criteria. The first column shows the GME certification results using the witness from Eq. (45) in Ref. [4]. The second column shows certification results using the witness in Eq. (22) of Ref. [7]. The third column reports GME/k-inseparability certified by our criteria with all terms in Eq. (2) measured. The fourth column shows results from our criteria with only the stabilizer generators measured, and all $|\langle S_iS_j\rangle|$ in Eq. (2) lower bounded by the dual SDP in Sec. II C. The last column gives the fidelities with the ideal graph states, calculated from the full simulated density matrices.

Supplementary Table S5: Tree-graph states

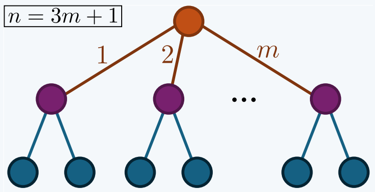

n	[4]'s witness	[7]'s witness	Our criteria	Our criteria (SDP)	Fidelity
4	GME	GME	GME	GME	0.846
7	/	GME	3-insep	4-insep	0.729
10	/	5-insep	5-insep	5-insep	0.630

Comparison of certified multipartite entanglement in simulated n-qubit tree-graph states using different criteria. The underlying graphs have a root of degree m, with each intermediate vertex branching into two leaves, giving a total of $n = 3m+1$ qubits. The first column shows GME certification results using the witness from Eq. (45) in Ref. [4]. The second column shows certification results using the witness in Eq. (15) of Ref. [7]. The third column reports GME/k-inseparability certified by our criteria with all terms in Eq. (2) measured. The fourth column shows results from our criteria with only the stabilizer generators measured, and all $|\langle S_iS_j\rangle|$ in Eq. (2) lower bounded by the dual SDP in Sec. II C. The last column shows fidelities with the ideal graph states, calculated from the full simulated density matrices.

[1] Géza Tóth and Otfried Gühne, *Detecting Genuine Multipartite Entanglement with Two Local Measurements,* Phys. Rev. Lett. **94**, 060501 (2005), arXiv:quant-ph/0405165.

[2] James O'Sullivan, Kevin Reuer, Aleksandr Grigorev, Xi Dai, Alonso Hernández-Antón, Manuel H. Muñoz-Arias, Christoph Hellings, Alexander Flasby, Dante Colao Zanuz, Jean-Claude Besse, Alexandre Blais, Daniel Malz, Christopher Eichler, and Andreas Wallraff, *Deterministic generation of two-dimensional multi-photon cluster states,* Nat. Commun. **16**, 5505 (2025), arXiv:2409.06623.

[3] Here and throughout the paper, we use the term *(local) measurement settings* to refer to the set of measurement-basis adjustments that an experiment must implement to measure all required observables, following the definition in Refs. [1, 4]. For example, measuring $X^{\otimes n} = (HZH)^{\otimes n}$ and $Z^{\otimes n}$ requires two measurement settings, whereas measuring $X \otimes Z \otimes \mathbb{1}$ and $\mathbb{1} \otimes Z \otimes X$ requires only one setting. Although this definition is the most commonly used one in the literature, the meaning of *measurement settings* can vary depending on the experimental platforms. For instance, in optical photonic experiments, it may refer to the number of combinations of basis states in local measurement bases (see, e.g., Ref. [52]). In contrast, in microwave photonic systems (see, e.g., Refs. [2, 53–55]), all measurement outcomes are obtained within a single measurement setting via heterodyne detection, with observables reconstructed through data post-processing.

[4] Géza Tóth and Otfried Gühne, *Entanglement detection in the stabilizer formalism,* Phys. Rev. A **72**, 022340 (2005), arXiv:quant-ph/0501020.

[5] Philip Thomas, Leonardo Ruscio, Olivier Morin, and Gerhard Rempe, *Efficient generation of entangled multiphoton graph states from a single atom,* Nature **608**, 677–681 (2022), arXiv:2205.12736.

[6] Philip Thomas, Leonardo Ruscio, Olivier Morin, and Gerhard Rempe, *Fusion of deterministically generated photonic graph states,* Nature **629**, 567–572 (2024), arXiv:2403.11950.

[7] You Zhou, Qi Zhao, Xiao Yuan, and Xiongfeng Ma, *Detecting multipartite entanglement structure with minimal resources,* npj Quantum Inf. **5**, 83 (2019), arXiv:1904.05001.

[8] Asher Peres, *Separability Criterion for Density Matrices,* Phys. Rev. Lett. **77**, 1413 (1996), arXiv:quant-ph/9604005.

[9] Michał Horodecki, Paweł Horodecki, and Ryszard Horodecki, *Separability of mixed states: necessary and sufficient conditions,* Phys. Lett. A **223**, 25 (1996), arXiv:quant-ph/9605038.

[10] Bastian Jungnitsch, Tobias Moroder, and Otfried Gühne, *Taming Multiparticle Entanglement,* Phys. Rev. Lett. **106**, 190502 (2011), arXiv:1010.6049.

[11] Bastian Jungnitsch, Tobias Moroder, and Otfried Gühne, *Entanglement witnesses for graph states: General theory and examples,* Phys. Rev. A **84**, 032310 (2011), arXiv:1106.1114.

[12] Marius Paraschiv, Nikolai Miklin, Tobias Moroder, and Otfried Gühne, *Proving genuine multiparticle entanglement from separable nearest-neighbor marginals,* Phys. Rev. A **98**, 062102 (2018), arXiv:1705.02696.

[13] Otfried Gühne, Chao-Yang Lu, Wei-Bo Gao, and Jian-Wei Pan, *Toolbox for entanglement detection and fidelity estimation,* Phys. Rev. A **76**, 030305 (2007), arXiv:0706.2432.

[14] Chao-Yang Lu, Xiao-Qi Zhou, Otfried Gühne, Wei-Bo Gao, Jin Zhang, Zhen-Sheng Yuan, Alexander Goebel, Tao Yang, and Jian-Wei Pan, *Experimental entanglement of six photons in graph states,* Nat. Phys. **3**, 91–95 (2007), arXiv:quant-ph/0609130.

[15] Konstantin Tiurev and Anders S. Sørensen, *Fidelity measurement of a multiqubit cluster state with minimal effort,* Phys. Rev. Res. **4**, 033162 (2022), arXiv:2107.10386.

[16] Steven T. Flammia and Yi-Kai Liu, *Direct Fidelity Estimation from Few Pauli Measurements,* Phys. Rev. Lett. **106**, 230501 (2011), arXiv:1104.4695.

[17] Marcus Huber, Florian Mintert, Andreas Gabriel, and Beatrix C. Hiesmayr, *Detection of High-Dimensional Genuine Multipartite Entanglement of Mixed States,* Phys. Rev. Lett. **104**, 210501 (2010), arXiv:0912.1870.

[18] Andreas Gabriel, Beatrix C. Hiesmayr, and Marcus Huber, *Criterion for k-separability in mixed multipartite systems,* Quantum Inf. Comput. **10**, 0829–0836 (2010), arXiv:1002.2953.

[19] Marcus Huber, Paul Erker, Hans Schimpf, Andreas Gabriel, and Beatrix C. Hiesmayr, *Experimentally feasible set of criteria detecting genuine multipartite entanglement in n-qubit Dicke states and in higher-dimensional systems,* Phys. Rev. A **83**, 040301(R) (2011), erratum Phys. Rev. A **84**, 039906 (2011), arXiv:1011.4579.

[20] Zhenhuan Liu, Yifan Tang, Hao Dai, Pengyu Liu, Shu Chen, and Xiongfeng Ma, *Detecting Entanglement in Quantum Many-Body Systems via Permutation Moments,* Phys. Rev. Lett. **129**, 260501 (2022), arXiv:2203.08391.

[21] Scott Aaronson, *Shadow tomography of quantum states,* in *Proceedings of the 50th Annual ACM SIGACT Symposium on Theory of Computing*, STOC 2018 (Association for Computing Machinery, New York, NY, USA, 2018) p. 325–338, arXiv:1711.01053.

[22] Hsin-Yuan Huang, Richard Kueng, and John Preskill, *Predicting many properties of a quantum system from very few measurements,* Nat. Phys. **16**, 1050–1057 (2020), arXiv:2002.08953.

[23] Steven J. van Enk and Carlo W. J. Beenakker, *Measuring $\mathrm{Tr}\rho^n$ on Single Copies of ρ Using Random Measurements,* Phys. Rev. Lett. **108**, 110503 (2012), arXiv:1112.1027.

[24] Tiff Brydges, Andreas Elben, Petar Jurcevic, Benoît Vermersch, Christine Maier, Ben P. Lanyon, Peter Zoller, Rainer Blatt, and Christian F. Roos, *Probing Rényi entanglement entropy via randomized measurements,* Science **364**, 260–263 (2019), arXiv:1806.05747.

[25] Andreas Elben, Benoît Vermersch, Christian F. Roos, and Peter Zoller, *Statistical correlations between locally randomized measurements: A toolbox for probing entanglement in many-body quantum states,* Phys. Rev. A **99**, 052323 (2019), arXiv:1812.02624.

[26] He Lu, Qi Zhao, Zheng-Da Li, Xu-Fei Yin, Xiao Yuan, Jui-Chen Hung, Luo-Kan Chen, Li Li, Nai-Le Liu, Cheng-Zhi Peng, Yeong-Cherng Liang, Xiongfeng Ma, Yu-Ao Chen, and Jian-Wei Pan, *Entanglement structure: Entanglement partitioning in multipartite systems and its experimental detection using optimizable witnesses,* Phys. Rev. X **8**, 021072 (2018), arXiv:1711.01784.

[27] Nicolai Friis, Oliver Marty, Christine Maier, Cornelius Hempel, Milan Holzäpfel, Petar Jurcevic, Martin B. Plenio, Marcus Huber, Christian Roos, Rainer Blatt, and Ben Lanyon, *Observation of Entangled States of a Fully Controlled 20-Qubit System,* Phys. Rev. X **8**, 021012 (2018), arXiv:1711.11092.

[28] Giorgos Stamatelatos and Pavlos S. Efraimidis, *Lexicographic Enumeration of Set Partitions,* arXiv:2105.07472 [cs.DM] (2021).

[29] Jack Edmonds, *Paths, Trees, and Flowers,* Can. J. Math. **17**, 449–467 (1965).

[30] Silvio Micali and Vijay V. Vazirani, *An $o(\sqrt{|v|} \cdot |e|)$ algorithm for finding maximum matching in general graphs,* in *21st Annual Symposium on Foundations of Computer Science (sfcs 1980)* (1980) p. 17–27.

[31] Although the Micali-Vazirani algorithm is more efficient, the blossom algorithm is used more often in solving the maximum cardinality matching problem as it is easier to implement.

[32] Donald Knuth, *Art of Computer Programming, Volume 4A, The Combinatorial Algorithms, Part 1* (Pearson Deutschland, 2011).

[33] Karl Goldberg, Morris Newman, and Emilie V. Haynsworth, *Combinatorial Analysis,* in *Handbook of Mathematical Functions: With Formulas, Graphs, and Mathematical Tables*, Applied mathematics series, edited by Milton Abramowitz and Irene A. Stegun (Dover Publications, 1965) p. 824–825.

[34] Maarten Van den Nest, Jeroen Dehaene, and Bart De Moor, *Graphical description of the action of local clifford transformations on graph states,* Phys. Rev. A **69**, 022316 (2004), arXiv:quant-ph/0308151.

[35] Marc Hein, Wolfgang Dür, Jens Eisert, Robert Raussendorf, Maarten Van den Nest, and Hans. J. Briegel, *Entanglement in graph states and its applications,* Proceedings of the International School of Physics "Enrico Fermi" **162**, 115–218 (2005), arXiv:quant-ph/0602096.

[36] Stephen Boyd and Lieven Vandenberghe, *Convex Optimization*, 7th ed. (Cambridge University Press, Cambridge, U.K., 2004).

[37] John Watrous, *The Theory of Quantum Information* (Cambridge University Press, Cambridge, U.K., 2018).

[38] Sara Bartolucci, Patrick Birchall, Hector Bombin, Hugo Cable, Chris Dawson, Mercedes Gimeno-Segovia, Eric Johnston, Konrad Kieling, Naomi Nickerson, Mihir Pant, Fernando Pastawski, Terry Rudolph, and Chris Sparrow, *Fusion-based quantum computation,* Nat. Commun. **14**, 912 (2023), arXiv:2101.09310.

[39] Robert Raussendorf and Hans J. Briegel, *A One-Way Quantum Computer,* Phys. Rev. Lett. **86**, 5188–5191 (2001), arXiv:quant-ph/0010033.

[40] Michael Varnava, Daniel E. Browne, and Terry Rudolph, *Loss Tolerance in One-Way Quantum Computation via Counterfactual Error Correction,* Phys. Rev. Lett. **97**, 120501 (2006), arXiv:quant-ph/0507036.

[41] Johannes Borregaard, Hannes Pichler, Tim Schröder, Mikhail D. Lukin, Peter Lodahl, and Anders S. Sørensen, *One-Way Quantum Repeater Based on Near-Deterministic Photon-Emitter Interfaces,* Phys. Rev. X **10**, 021071 (2020), arXiv:1907.05101.

[42] Fei Shi, Lin Chen, Giulio Chiribella, and Qi Zhao, *Entanglement Detection Length of Multipartite Quantum States,* Phys. Rev. Lett. **134**, 050201 (2025), arXiv:2401.03367.

[43] Robert Henry Dicke, *Coherence in Spontaneous Radiation Processes,* Phys. Rev. **93**, 99 (1954).

[44] Octavio Castaños, Ramón López-Peña, Jorge G. Hirsch, and Enrique López-Moreno, *Classical and quantum phase transitions in the Lipkin-Meshkov-Glick model,* Phys. Rev. B **74**, 104118 (2006).

[45] Robert Prevedel, G. Cronenberg, M. S. Tame, Mauro Paternostro, Philip Walther, Myungshik Kim, and Anton Zeilinger, *Experimental Realization of Dicke States of up to Six Qubits for Multiparty Quantum Networking,* Phys. Rev. Lett. **103**, 020503 (2009), arXiv:0903.2212.

[46] Géza Tóth, *Multipartite entanglement and high-precision metrology,* Phys. Rev. A **85**, 022322 (2012), arXiv:1006.4368.

[47] Christopher Eichler, Deniz Bozyigit, Christian Lang, Lars Steffen, Johannes Fink, and Andreas Wallraff, *Experimental State Tomography of Itinerant Single Microwave Photons,* Phys. Rev. Lett. **106**, 220503 (2011), arXiv:1011.6668.

[48] Christopher Eichler, Deniz Bozyigit, and Andreas Wallraff, *Characterizing quantum microwave radiation and its entanglement with superconducting qubits using linear detectors,* Phys. Rev. A **86**, 032106 (2012), arXiv:1206.3405.

[49] Marcus P. Da Silva, Deniz Bozyigit, Andreas Wallraff, and Alexandre Blais, *Schemes for the observation of photon correlation functions in circuit QED with linear detectors,* Phys. Rev. A **82**, 043804 (2010), arXiv:1004.3987.

[50] Manuel H. Muñoz-Arias, GitHub. Retrieved from `https://github.com/manuelmz/sequential_gen_photonic_graph_states` on January 27, 2026.

[51] J. Frédéric Bonnans and Alexander Shapiro, *Perturbation Analysis of Optimization Problems*, Springer Series in Operations Research and Financial Engineering (Springer New York, 2000).

[52] Nicolai Friis, Giuseppe Vitagliano, Mehul Malik, and Marcus Huber, *Entanglement Certification From Theory to Experiment,* Nat. Rev. Phys. **1**, 72–87 (2019), arXiv:1906.10929.

[53] Jean-Claude Besse, Kevin Reuer, Michele C. Collodo, Arne Wulff, Lucien Wernli, Adrian Copetudo, Daniel Malz, Paul Magnard, Abdulkadir Akin, Mihai Gabureac, Graham J. Norris, J. Ignacio Cirac, Andreas Wallraff, and Christopher Eichler, *Realizing a deterministic source of multipartite-entangled photonic qubits,* Nat. Commun. **11**, 4877 (2020), arXiv:2005.07060.

[54] Vinicius S. Ferreira, Gihwan Kim, Andreas Butler, Hannes Pichler, and Oskar Painter, *Deterministic generation of multidimensional photonic cluster states with a single quantum emitter,* Nat. Phys. **20**, 865–870 (2024), arXiv:2206.10076.

[55] Yoshiki Sunada, Shingo Kono, Jesper Ilves, Takanori Sugiyama, Yasunari Suzuki, Tsuyoshi Okubo, Shuhei Tamate, Yutaka Tabuchi, and Yasunobu Nakamura, *Efficient Tomography of Microwave Photonic Cluster States,* arXiv:2410.03345 [quant-ph] (2024).
